# EFFECTS OF FK506 ON THE HEALING OF DIAPHYSEAL, CRITICAL SIZE DEFECTS IN THE RAT FEMUR

**DOI:** 10.22203/eCM.v040a10

**Published:** 2020-10-06

**Authors:** R.E. De la Vega, M.J. Coenen, S.A. Müller, C.V. Nagelli, N.P. Quirk, C. Lopez de Padilla, C.H. Evans

**Affiliations:** 1 Musculoskeletal Gene Therapy Research Laboratory, Rehabilitation Medicine Research Center, Mayo Clinic, Rochester, MN, USA; 2 Department cBITE and Department IBE, MERLN - Institute for Technology-Inspired Regenerative Medicine, Maastricht University, Maastricht, the Netherlands; 3 Orthopaedic Department, University of Basel, Basel, Switzerland

**Keywords:** Immunosuppression, bone healing, gene therapy, adenovirus, rat, BMP-2

## Abstract

There is much interest in understanding the influence of the immune system on bone healing, including a number of reports suggesting a beneficial effect of FK506 (tacrolimus) in this regard. The influence of FK506 in a rat, femoral, critical size defect was examined using locally implanted, recombinant, human (rh) BMP-2 and adenovirally-transduced, autologous, adipose-derived mesenchymal stromal cells (AD-MSCs) expressing BMP-2. FK506 was delivered systemically using an implanted osmotic pump. Empty defects and those implanted with unmodified AD-MSCs did not heal in the presence or absence of FK506. Defects treated with rhBMP-2 healed with a large callus containing thin cortices and wispy trabeculae; this, too, was unaffected by FK506. A third of defects implanted with adenovirally-transduced AD-MSCs healed, but this improved to 100 % in the presence of FK506. New bone formed in response to BMP-2 synthesised endogenously by the genetically modified cells had a slimmer callus than those healed by rhBMP-2, with improved cortication and advanced reconstitution of marrow. These results suggest that FK506 may have had little effect on the intrinsic biology of bone healing, but improved healing in response to adenovirally-transduced cells by inhibiting immune responses to the first-generation adenovirus used here. Because the genetically modified cells produced bone of higher quality at far lower doses of BMP-2, this approach should be explored in subsequent research.

## Introduction

Recombinant, human bone morphogenetic protein-2 (rhBMP-2) is the active ingredient of the product Infuse (Medtronic, Minneapolis, MN, USA) that has FDA approval for use in spinal fusion, certain dental applications and open tibial fractures. However, it has wide off-label application in multiple clinical settings where it is necessary to grow bone, including long bone fractures and segmental defects. BMP-2 is applied on a collagen sponge at mg doses which increase costs and are associated with adverse side effects (James *et al.*, 2016). Moreover, its efficacy and cost effectiveness have been questioned (Garrison *et al.*, 2010).

Gene transfer has been suggested as a more effective way of delivering BMP-2 to sites of osseous lesion, and considerable success has been reported when using preclinical models (Evans and Huard, 2015). The authors’ laboratory has extensive experience in this context using a rat, 5 mm, critical size, femoral, diaphyseal defect. During studies on the healing of such defects by gene transfer, it was noted that immunosuppression with FK506 alone or in combination with SEW2871 enhanced bone healing (De la Vega *et al.*, 2018; Liu *et al.*, 2015). In these experiments, a first generation adenovirus vector was used to transfer bone morphogenetic protein-2 (BMP-2) cDNA into the osseous defects by means of transduced xenogeneic (Liu *et al*., 2015) or allogeneic (De la Vega *et al*., 2018) muscle grafts. FK506, in the form of the drug tacrolimus, is used as an immunosuppressant in transplant recipients where it blocks T-cell function by inhibiting calcineurin (Thomson *et al.*, 1995). This provides a potential mechanism of action for stimulating bone healing.

However, the literature on the role of T-cells in bone healing is contradictory. This is best illustrated in studies using Rag1^−/−^ mice that lack functional lymphocytes; one publication reports accelerated fracture healing (Toben *et al.*, 2011) while another reports impaired fracture healing (Nam *et al.*, 2012). NOD/SCID mice lacking functional macrophages, natural killer cells, dendritic cells and lymphocytes show impaired bone healing (Rapp *et al.*, 2016) while bone healing is enhanced in mice deficient in γ/δ T-cells (Colburn *et al.*, 2009).

Reinke *et al.* (2013) studied healing of femoral defects in mice from which CD8^+^ lymphocytes had been depleted with antibodies or augmented by adaptive transfer. There was no difference in bone mineral density or the ratio of bone volume to total volume between wild-type and CD8-depleted mice, but these values were reduced in CD8^+^ augmented animals. Data from human patients studied by the same authors suggest that impaired healing of tibial plateau fractures was associated with the presence of elevated numbers of CD8^+^ memory T-cells (Reinke *et al*., 2013).

Another potential mechanism through which FK506 could enhance bone healing is provided by the observation that FK506 enhances BMP-2 signal transduction (Kugimiya *et al.*, 2005). Within the cell, FK506 binds to FK binding protein-12 (FKBP12), a molecule that prevents the type II BMP receptor from phosphorylating the type I BMP receptor, thereby blocking signal transduction *via* the Smad 1/5/8 pathway (Spiekerkoetter *et al.*, 2013). FK506 relieves the inhibition imposed by FKBP12. A number of independent studies using different mesenchymal cell lines have demonstrated the ability of FK506 to enhance differentiation along osteoblastic lineages *in vitro* in response to BMP-2 (Darcy *et al.*, 2012; Kugimiya *et al*., 2005; Tang *et al.*, 2002). Of relevance to the endochondral route to bone formation, FK506 also enhances the differentiation of mesenchymal progenitor cells into chondrocytes in the presence of BMP-2 (Nishigaki *et al.*, 2002; Tateishi *et al.*, 2007). These circumstances encourage the evaluation of FK506 as an agent of enhanced bone healing, despite knowing that systemically delivered FK506 induces osteopenia and osteoporosis in organ transplant recipients (Kulak *et al.*, 2012).

Several studies confirm that FK506 promotes the formation of bone ectopically after implantation of BMPs on a collagenous scaffold. Voggenreiter *et al.* (2000), for instance, noted a marked stimulation of osteogenesis by FK506 in a rat model of ectopic bone formation using demineralised bone matrix implanted intra-muscularly. In similar types of experiments, Kaihara *et al.* (2002) and Sangadala *et al.* (2019) used FK506 to enhance ectopic bone formation after the implantation of rhBMP-2 on collagen sponges implanted intra-muscularly and sub-cutaneously, respectively. Nevertheless, later research from Voggenreiter *et al.* (2005) found no effect of FK506 on the healing of tibial fractures in rats. Possible insight into this discrepancy is provided by Hurtgen *et al.* (2017), who studied the effects of FK506 on healing of tibial osteotomies with or without the concomitant injury of the adjacent tibialis anterior muscle. FK506 only improved healing of the tibia in animals where the adjacent muscle was also injured. In the absence of muscle injury, there was no effect of FK506 on healing of the tibia.

In view of the highly confusing literature, summarised above, the issue was revisited using a validated, critical size, femoral defect model in Fischer rats to compare directly the effects of FK506 on bone healing induced by rhBMP-2 or genetically modified, adipose-derived mesenchymal stromal cells. The latter were selected because they are commonly used in studies of *ex vivo* gene therapy for bone healing.

## Materials and Methods

### Study Design

A 5 mm, mid-femoral defect was created in the right hind limb of 80 male, 16-week-old Fischer F344 rats and stabilised with an internal, polyacetal, custom-made plate. Rats were randomly divided into 8 experimental groups (*n* = 10) (Table 1). Group size was based upon the need to provide 2 animals for histology and 8 animals for mechanical testing. Sample size for the latter is based on historical laboratory data using statistical power of 80 % (β = 0.20). Power analysis revealed that *n* = 8 animals per group for biomechanical testing would allow detection of significant differences between groups, based upon means and standard deviations from intact femora, using a two-sided α error of 0.05. These were determined using the statistical analysis software Stata, version 11.2 (StataCorp, College Station, TX, USA). Defects remained empty, or received rhBMP-2, adipose-derived mesenchymal stromal cells (AD-MSCs), or genetically modified, adenovirus (AV)-transduced AD-MSCs expressing BMP-2 (AV.AD-MSCs). FK506 was delivered as a continuous systemic infusion using subcutaneous osmotic pumps at a rate of 1 mg/kg/d for 4 weeks post-surgery in order to minimise animal handling. Bone healing was radiographically monitored using an x-ray cabinet. Animals were euthanised 8 weeks post-surgery, the femora harvested and the bone healing response assessed by micro-computed tomography (μCT) (*n* = 10/group), histology (*n* = 2/group) and biomechanical testing (*n* = 8/group). Femora that did not heal radiologically were not subject to biomechanical testing.

### Animals

Male Fischer F344 rats were purchased from Charles River Laboratories (Wilmington, MA, USA). All rats were housed at the Mayo Clinic animal care facility (Rochester, MN, USA) with 12 h light cycles and were given food and water *ad libitum*. Animal care and experimental protocols were followed in accordance with the NIH guidelines and approved by the Mayo Clinic Institutional Animal Care and Use Committee (#A00001567–16). All animals undergoing survival surgery received cefazolin at 50 mg/kg for antibiotic prophylaxis and buprenorphine slow-release at 1 mg/kg as analgesic 30 min before the start of the surgical procedure. A second dose of buprenorphine was administered 3 d after the procedure. After surgery, animals were allowed to move freely within their cages.

### Primary cell culture isolation and reagents

AD-MSCs were harvested from the adipose tissue of donor F344 rats (14 weeks of age) and expanded *in vitro*. F344 rats were used because they are syngeneic, thus minimising the possibility of an immune reaction to donor cells. Briefly, in sterile fashion, adipose tissue was harvested from the epididymal, perirenal and intestinal fat, minced in small pieces, washed in phosphate buffered saline (PBS; Lonza, Basel, Switzerland), digested in 0.2 % collagenase A type I (Sigma-Aldrich, St. Louis, MO, USA) for 60 min at 37 °C and the vascular stromal fraction (VSF) separated by a 100 μm strainer (ThermoFisher, Waltham, MA, USA) followed by centrifugation at 400 ×*g* for 10 min. The VSF was seeded and expanded in Dulbecco’s modified Eagle’s medium (DMEM; ThermoFisher) with 10 % foetal bovine serum (FBS; ThermoFisher), penicillin 100 Units/mL and 100 μg/mL streptomycin (ThermoFisher). After one week in the same culture conditions, adherent cell colonies were recovered using TrypLE Express (ThermoFisher) and further expanded in the same growth medium. All experiments involving AD-MSCs were performed with cells in passages 2–4.

FK506 (Cayman Chemical, Ann Arbor, MI, USA) was reconstituted in a 50 % dimethyl sulphoxide (DMSO; Sigma-Aldrich) and 15 % ethanol (ThermoFisher) solution to a final concentration of 5 mg/mL and stored at − 80 °C. Recombinant human BMP-2 (rhBMP-2; Medtronic, Fridley, MN, USA) was reconstituted in sterile water to a final concentration of 0.11 mg/mL. Reconstituted rhBMP-2 (11 μg; 100 μL) was added to a 12 × 7 × 5 mm collagen sponge (Helistat; Integra LifeSciences, Plainsboro, NJ, USA) at the beginning of surgery.

### Transduction and characterisation of genetically modified cells

AD-MSCs were genetically modified to express BMP-2 using a first-generation, serotype 5, adenovirus (ΔE1, ΔE3) containing human BMP-2 cDNA (Ad. BMP-2) (Gouze *et al.*, 2004) by rapid transduction. Briefly, AD-MSCs were expanded to sufficient numbers and lifted from the culture vessel. Cells were pelleted and re-suspended in serum-free DMEM containing Ad.BMP-2 at a dose of 2,500 viral particles per cell (vp/cell) to a final concentration of 5 × 10^6^ cells/mL. To expedite and enhance transduction efficiency, cells were spinoculated in the virus solution at 2,000 ×*g* for 20 min at 37 °C (Meeting abstract: Hauser-Schinhan *et al.*, 2015, S-252. DOI: 10.1089/ten.tea.2015.5000.abstracts). Virus-containing supernatant was removed immediately after centrifuging. The cell pellet was resuspended and washed in phosphate buffered saline to remove free viral particles. The cell suspension was centrifuged again and the pelleted, genetically modified, AD-MSCs encapsulated in fibrin (TISSEEL; Baxter, Deerfield, IL, USA) at a density of 4 × 10^6^ cells/gel, using a 96-well plate (Corning Inc., Corning, NY, USA) as a template to obtain a 6 mm in diameter by 5 mm in height cylindrical gel. Non-transduced AD-MSCs were encapsulated in fibrin in the same fashion. Fibrin gels were cultured in ultra-low attachment 24-well plates (Corning), with regular growth medium supplemented with 1.5 mg/mL of the plasmin inhibitor aminocaproic acid (Sigma-Aldrich), and medium changed every other day. Three biological replicates per group were used. BMP-2 levels were determined on the collected medium every 48 h using the human BMP-2 DuoSet ELISA kit (R&D Systems, Minneapolis, MN, USA). Fibrin gels were surgically implanted within the critical size defect 2 d after encapsulation and the medium from each individual well was stored for subsequent ELISA measurement of BMP-2 pre-implantation levels.

### Surgical model

F344 rats at 16 weeks of age were used for surgery as described previously (De la Vega *et al*., 2018). Briefly, a 5 mm, critical-sized, mid-femoral defect was created in the right hind limb of each rat. Under sterile conditions and isoflurane anaesthesia, a 4 cm incision was made on the lateral thigh. The intermuscular septum with respect to the femur was dissected to expose the femoral diaphysis. The soft tissues surrounding the bone, including the periosteum, were preserved. Using a polyacetal plate (Special Designs, La Vernia, TX, USA) as a guide, 4 holes were drilled along the mid-diaphysis using a 0.79 mm drill bit (RISystem AG, Davos Platz, Switzerland). The plate was then secured carefully to the femur using four hand-driven 0.9 mm threaded K-wires (MicroAire Surgical Instruments, Charlottesville, VA, USA) cut to a length of 11 mm, which allowed the construct to act as a locking plate. A 5 mm osteotomy was created equidistant from each of the inner wires marked by a small notch in the plate using a 0.44 mm Gigli wire saw (RISystem AG). After completion of the osteotomy, the bone fragment was removed and the site was irrigated with normal saline, and the defect was left empty, given untreated collagen sponge or received one of the treatments summarised in Table 1. A soft tissue pouch was created using the adjacent muscles to ensure the implanted treatments would stay within the defect. The wound was closed in layers with 4–0 Vicryl and the incision site closed using 9 mm wound clips. A subcutaneous pouch was immediately created in the right lumbar region of animals receiving FK506. A 30 mm skin incision was made above the right iliac crest and the skin was gently separated from the underlying spinal muscles by cephalic blunt dissection. An osmotic pump (#2ML4; ALZET Osmotic Pumps, Cupertino, CA, USA) containing FK506 at a concentration of 5 mg/mL was inserted with the opening on the cephalic orientation and secured with a subcutaneous 4–0 Vicryl stitch. The skin incision was closed using 9 mm wound clips. Animals recovered from anaesthesia in a heated chamber at 32 °C and were followed for 8 weeks after surgery when the treated femora and the contralateral, unoperated femora were harvested for analysis. Wound clips were removed after 10 d under anaesthesia while the first radiograph was obtained. Osmotic pumps remained in the animals receiving FK506 for the duration of the experiment.

### Radiographic monitoring

*In vivo* bone healing was monitored by imaging the right femur using a digital x-ray cabinet system (MX-20, Faxitron Bioptics, Tucson, AZ, USA) under general anaesthesia with isoflurane. Rats were ventrally positioned inside the cabinet with the hind limbs abducted at a 90° angle from the body. Radiographs were obtained at 42 kV energy and 10 s exposure time on days 10, 28, 42 and 56. Two observers analysed the images for fractures, failure of fixation, bone formation and bridging of the defect. Healing of the defect was defined as the presence of a bony callus spanning the length of the defect that bridged the four cortices observed on the lateral x-ray.

### μCT analysis

All harvested femora underwent μCT scanning. The architecture of the newly formed bone in the rat defects was examined with a desktop μCT imaging system (vivaCT 40, Scanco Medical AG, Bassersdorf, Switzerland) equipped with a 10 mm focal spot microfocus X-ray tube. Femoral defects were scanned using a 20 μm isotropic voxel size, at 55 kV energy, 200 ms integration time, with approximately 500 slices per specimen. The slices were converted to DICOM format using the vivaCT 40 software and then imported for analysis using AnalyzePro software version 1.0 (Biomedical Imaging Resource, Mayo Clinic, MN, USA). Bone structure was segmented by automated thresholding, separating the bone from the surrounding tissue, and common bone morphometric indices were calculated using the Bone Microarchitecture Analysis module in the software. A central, 4 mm (200 slice) region of each defect, defined as 2 mm proximal and 2 mm distal to the mid-point between the inner pins of the plate was used to exclude any pre-existing intact cortical bone. Total volume of the bony callus (TV, mm^3^), bone volume (BV, mm^3^), and BV/TV fraction (%) were calculated from μCT images.

### Histology

Two of the treated femora per group and all femora that failed to heal were fixed in 10 % neutral-buffered formalin (ThermoFisher) for 72 h. Samples were decalcified in 20 % formic acid (Sigma-Aldrich) with constant agitation at room temperature for 72 h. The endpoint of decalcification was determined radiographically. Specimens were processed as described before (De la Vega *et al*., 2018). Briefly, decalcified specimens were dehydrated in a series of graded alcohols, embedded in paraffin wax and 5 μm thick sections obtained using an automatic microtome (HM 355S, ThermoFisher). Sections were mounted on glass slides (Superfrost Plus, ThermoFisher) and standard protocols followed for staining with haematoxylin & eosin (H&E) and safranin O/fast green (SOFG). Images were acquired using an inverted microscope with a motorised stage (Olympus IX83, Olympus, Waltham, MA, USA) coupled to the cellSens Dimension Desktop software (version 2.2; Olympus). Images were analysed for bone formation, bone remodelling, cartilage tissue, fibrous tissue, cellular infiltrate and bone marrow within the defect.

### Biomechanical testing

Harvested femora of healed bone defects were cleaned of soft tissue, the fixation hardware removed and subsequently wrapped in 0.9 % saline soaked gauze and frozen at −20 °C until testing. Frozen femora underwent gradual thawing at 4 °C for 24 h before testing. Biomechanical properties of the healed bone defects were obtained by conducting torsional testing to failure in a blinded fashion. Because restoration of defect integrity is required for testing, femora from animals that did not heal the defect as evidenced by radiographs were not subject to torsional testing. Groups 6–8 had *n* = 8 specimens, while group 5 had *n* = 3 specimens, and groups 1–4 had no healed specimens for testing. Each experimental specimen had its contralateral, intact femur tested as a control. Prior to testing, the ends of each femur were potted in polymethylmethacrylate within a square metal fixture to fix the specimens in a reliable fashion. A custom-made materials-testing system for small animals (Mayo Clinic, Rochester, MN) was used to conduct the torsional testing. This machine interfaces with a LabVIEW software (National Instruments, Austin, TX, USA) designed to output displacement and load (Model RTS-200, Transducer Techniques LLC, Temecula, CA, USA). Bones were tested to failure under deformation control at a constant deformation rate of 5 rad/min. The torque and displacement was used to calculate strength to failure (Nm) and torsional stiffness (Nm/rad).

### Statistical analysis

Statistical analysis was performed using the software Stata v11.2. Healing data from radiographs comparing one group against its homonym receiving FK506 was performed using the Pearson *χ*^*2*^ test. Data obtained from μCT analysis were compared using a one-way ANOVA and Bonferroni *post-hoc* analysis. Biomechanical testing data were analysed with the Kruskal-Wallis test to confirm non-parametric differences between groups, and *p* values between treatment groups were obtained by the Mann-Whitney U test; a *p* value less than 0.05 was considered statistically significant.

## Results

### BMP-2 production by genetically modified cells

**Fig. 1.**
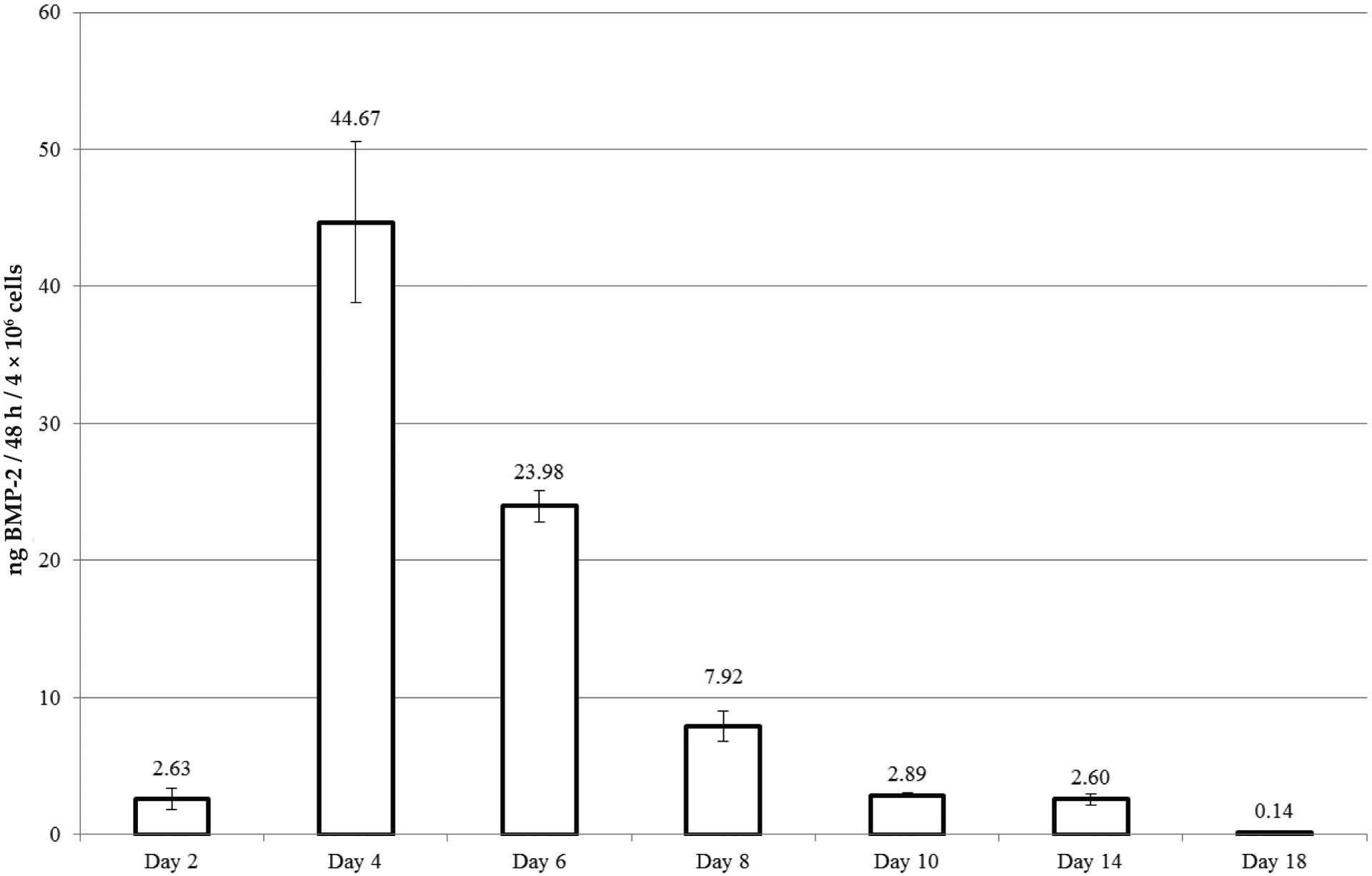
BMP-2 production by genetically modified cells encapsulated in fibrin. BMP-2 was quantified in the culture medium of fibrin-encapsulated AV.AD-MSCs using a commercially available ELISA kit specific for the human protein. Medium was collected and replenished every 48 h. Time course of BMP-2 production *in vitro* by AV.AD-MSCs fibrin gels (*n* = 3) with results shown as bars reflecting the mean and whisker plots representing the standard deviation.

Successful transduction of the AD-MSCs by adenovirus (AV.AD-MSCs) was confirmed by measuring human BMP-2 production by the encapsulated cells for up to 18 d after transduction. BMP-2 levels peaked on day 4 post-transduction at 44.67 ± 5.87 ng followed by a rapid decrease in production as shown in Fig. 1. There was no detectable BMP-2 in the medium after day 18, even though cell viability was confirmed until day 26 post-transduction using a live cell assay with fluorescein diacetate (data not shown) where an abundance of green fluorescent cells was observed.

Having confirmed sustained BMP-2 production by the genetically modified cells contained within fibrin (Fig. 1), fresh cultures were established. 2 d prior to surgery, these cells were encapsulated in fibrin and individual constructs maintained in culture medium. At the time of subsequent surgery, individual constructs were randomly selected for implantation in femoral defects without prior measurement of BMP-2 levels. After surgical implantation, BMP-2 levels were measured retrospectively by ELISA on the conditioned media. *In vivo* measurements were not made. Rats in group 5 that did not receive *in vivo* FK506 produced 2.3 ± 0.4 ng BMP-2 on day 2 after encapsulation and rats in group 6, that did receive *in vivo* FK506 produced 1.3 ± 0.4 ng BMP-2 at this time.

### Bone healing in the presence or absence of FK506

All animals tolerated the surgical procedure and were able to bear weight and mobilise the operated limb in the immediate post-operative period. One animal from group 5 was found to have a femoral fracture through the distal k-wire at 10 d and was euthanised according to protocol. The 79 remaining animals lived for 8 weeks without any incident.

**Fig. 2.**
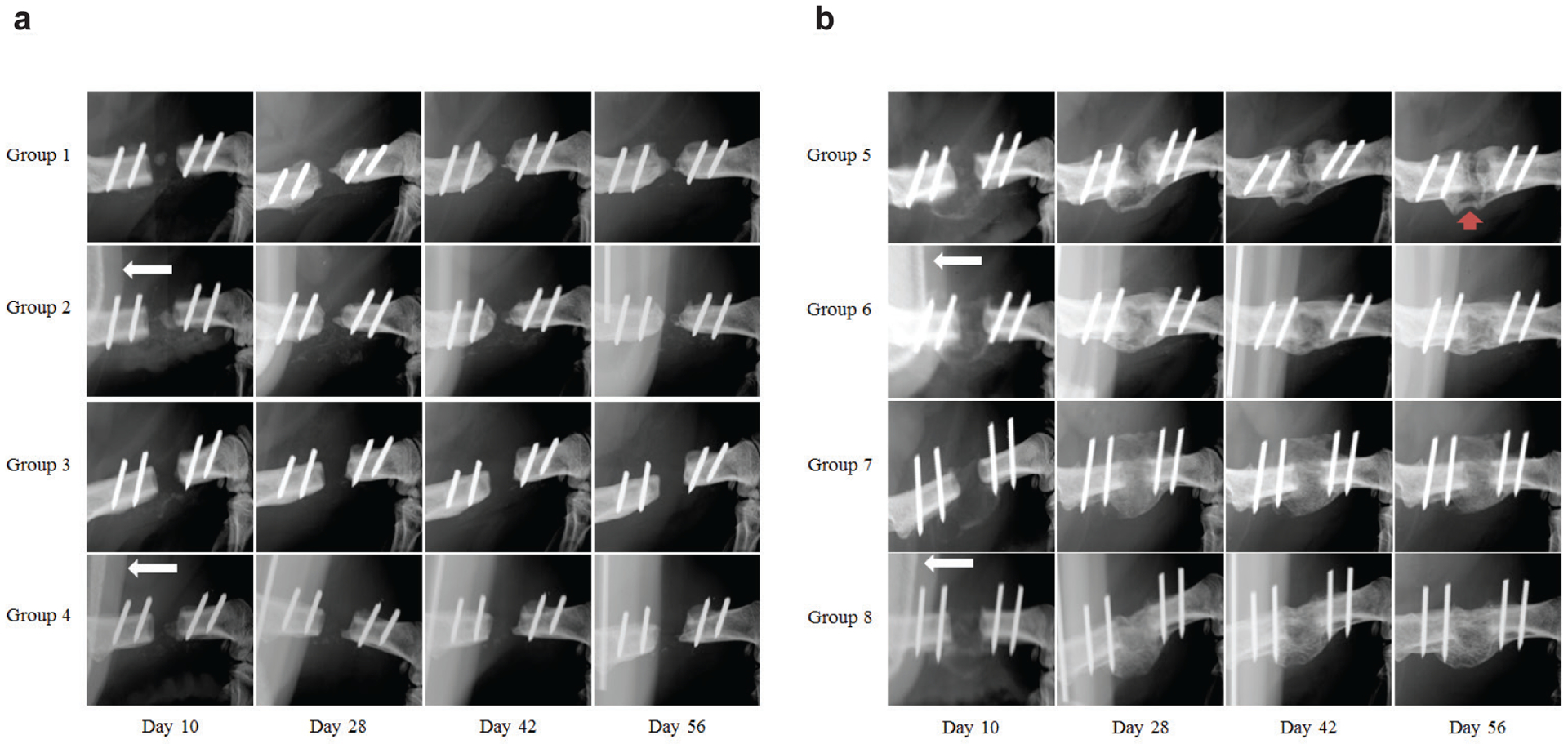
Radiographic time course of femoral defects with various treatments in the presence or absence of systemic FK506 treatment. (**a**) Radiographs of treatment groups in the absence of BMP-2. (**b**) Radiographs of treatment groups with either endogenous transgenic expression of BMP-2 (groups 5 and 6) or exogenous recombinant human BMP-2 (groups 7 and 8). White arrows in groups 2, 4, 6 and 8 depict the presence of the osmotic pump delivering FK506. Red arrow in group 5 shows failure to bridge completely and bone cyst-like structure. Only 3 of 9 defects in group 5 bridged.

In the absence of recombinant or transgenic BMP-2 there was no evidence of radiographic bone healing in any of the groups (groups 1–4), including those receiving the untreated collagen sponge (data not shown). New bone formation in these groups was present at the defect edges accompanied by periosteal reaction in all animals at day 28, only to result in sclerotic bony caps by week 8 without any noticeable differences between animals with and without FK506 (Fig. 2a).

Only animals receiving BMP-2 presented with radiographic bridging of the defect (Table 2). In all groups with BMP-2 (groups 5–8), either from transgene expression (groups 5 and 6) or as recombinant protein (groups 7 and 8), there was evidence of a bony callus being deposited and bridging as early as day 10 (Fig. 2b). Animals treated with rhBMP-2 showed consistent healing of the defect, characterised by a prominent callus extending outside the perimeter of the femur presenting as a more radiolucent bone than that of the naturally radiopaque cortical bone, independent of FK506 treatment (Fig. 2b, groups 7 and 8).

Only in animals treated with AV.AD-MSCs did FK506 influence the healing outcome. All 10 animals in group 6, which received FK506, healed the defect by 8 weeks while only 3 out of 9 animals in group 5, lacking FK506, healed (*p* = 0.002). New bone formation was abundant in AV.AD-MSCs treated animals, with a callus smaller than that of the rhBMP-2 treated animals and more confined to the defect area. Defects in group 6 had a denser and more uniform callus bridging the defect edges while most of defects in group 5 were noticeable for bone that failed to bridge, inconsistent callus size, and areas of radiolucency resembling bone cysts (Fig. 2b).

### Qualitative and quantitative bone healing microarchitecture

**Fig. 3.**
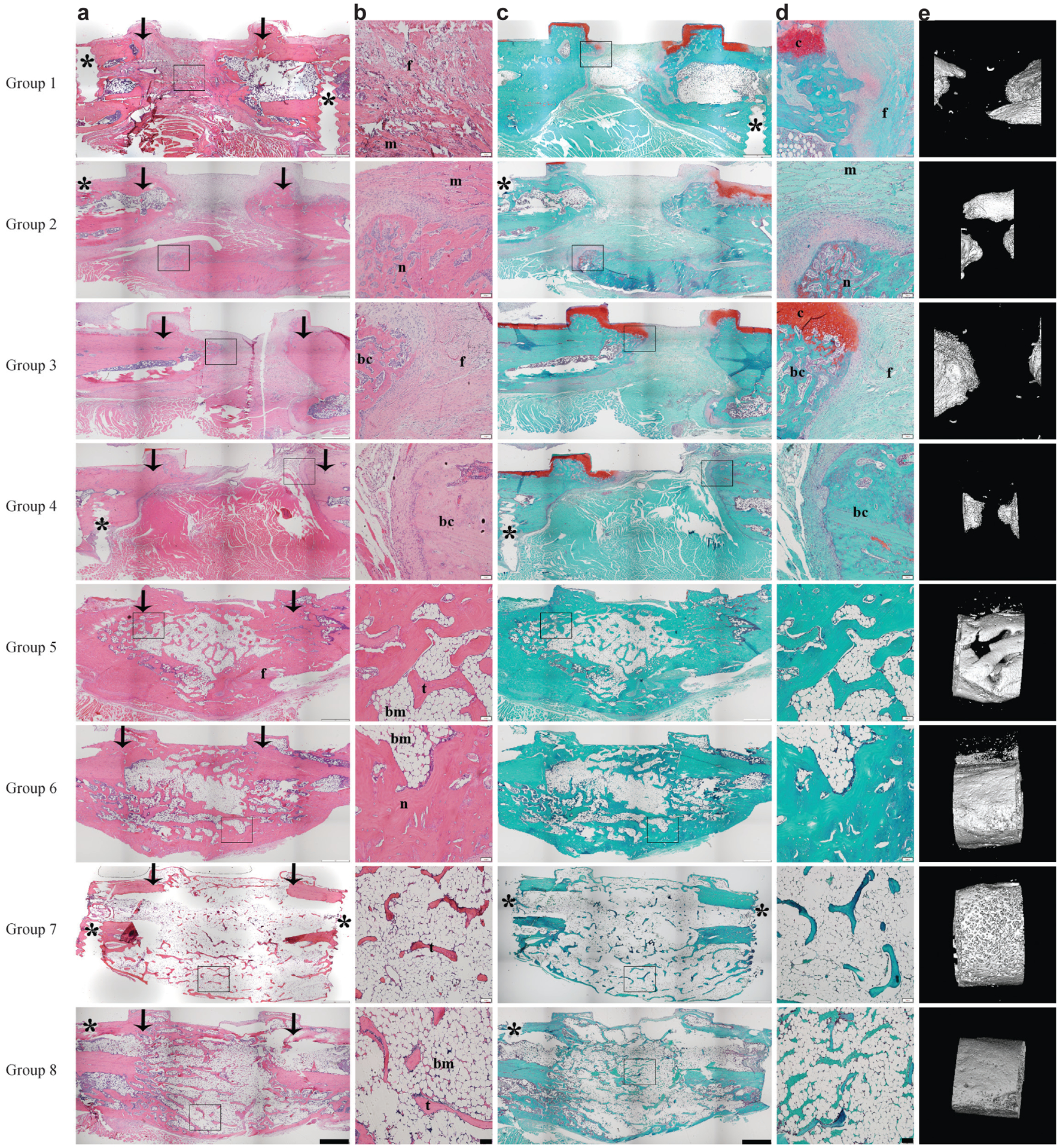
Histological appearance of defects 8 weeks after various treatments in the presence or absence of systemic FK506 treatment. Column (**a**) shows representatives images of sections stained with H&E at low magnification and stitched by the automated microscope software (bottom panel scale bar = 1 mm). Column (**b**) shows the boxed region in column (**a**) at higher magnification (bottom panel scale bar = 100 μm). Column (**c**) shows stitched low magnification images stained with SOFG (bottom panel scale bar = 1 mm). Column (**d**) shows the boxed region in column (**c**) at higher magnification (bottom panel scale bar = 100 μm). Column (**e**) shows 3D reconstructed μCT images depicting the extent of bone union. Asterisks in column (**a**) and (**c**) indicate the pin holes for the fixation plate. Arrows indicate the edge of the original defect. “f” denotes fibrous repair tissue, “m” denotes muscle tissue, “c” denotes cartilage tissue within the defect, “n” denotes new cortical bone formation, “bc” denotes the presence of the bony caps at the edge of the defect, “bm” denotes bone marrow, “t” denotes trabecular bone.

**Fig. 4.**
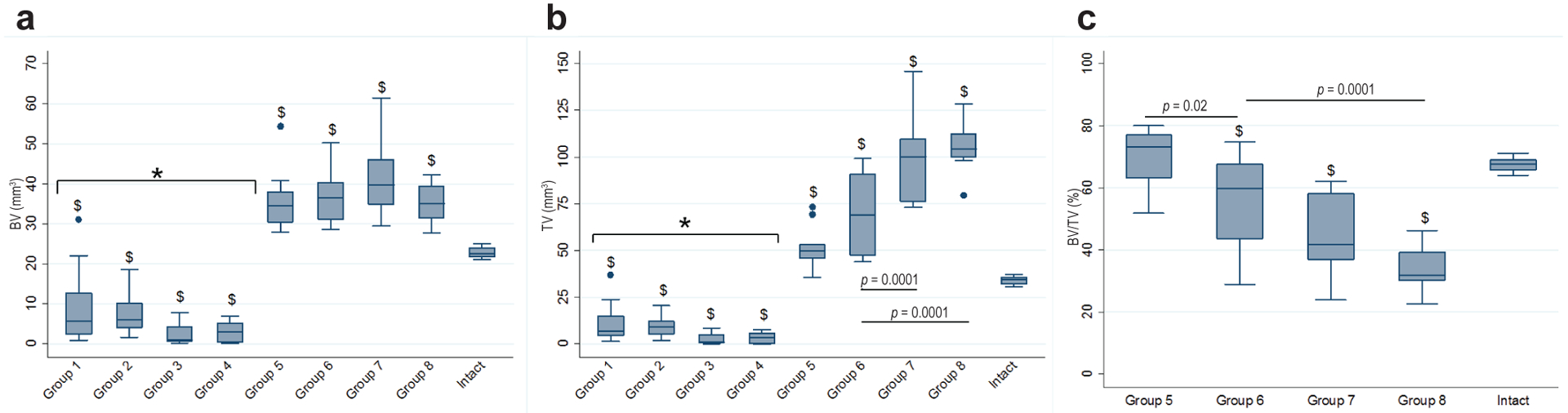
Volumes of the defects 8 weeks after various treatments in the presence or absence of systemic FK506 treatment. Treated and untreated femora were harvested from rats 8 weeks after surgery. The 4 mm central region of the defect was scanned using a desktop μCT scanner and common bone morphometric values obtained after automated contouring. (**a**) Bone volume (BV). (**b**) Total volume (TV). (**c**) Bone volume to total volume fraction (BV/TV). Statistical difference between groups is shown when *p* values are < 0.05. $ denotes statistical difference when compared to intact femora. * denotes statistical difference when compared to BMP-2 treated groups (groups 5–9). Brackets are used to group together several treatments in order to compare to a single treatment or other grouped treatments. Statistical difference in between specific groups is denoted by a horizontal line with the corresponding *p* value. Data are presented in box and whisker plots representing the interquartile range as a box, the median as the horizontal bar in the box, the upper and lower adjacent value as whiskers, and outside values as dots.

Histological analysis of the tissue filling the defect area of all animals in groups 1–4 revealed a mix of fibrous and muscle tissue, with some areas of cartilage formation abutting the fixation plate and intramembranous ossification around the bone defect stumps (Fig. 3a–d). μCT analysis of these defects showed there was also no difference in BV values in between groups 1–4 (Fig. 4a). All femora from these treatment groups had BV and TV significantly lower than those of the untreated, intact femora (*p* ≤ 0.001). Bone within the defect correlated with histology findings, with most of the bone being limited to the bony, sclerotic caps with the middle of the defect void of any hard tissue. Interestingly, both groups receiving AD-MSCs (3 & 4) presented with lower BV values than those of groups where the defects where left empty (1 & 2), but no statistical difference was found.

When defects were treated with BMP-2 (groups 5–8), BV and TV values were statistically higher than those without BMP-2 treatment (groups 1–4), and those of the intact femora (*p* ≤ 0.001) (Fig. 4a–b). There was no statistical difference in BV between any of the groups 5–8 except when compared to the intact femora, in which case all groups that received BMP-2 were found to have significantly higher BV values than the intact femora control (*p* = 0.0001). Differences between BMP-2 treatment groups were observed when comparing TV results. Group 6 was found to have significantly smaller TV than individual groups 7 and 8 (*p* = 0.0001). When grouped together, the TV of both the rhBMP-2 treated groups (groups 7–8) was statistically higher than the paired AV-AD-MSCs treated groups (groups 5–6) (*p* = 0.0001) (Fig. 4b) showing a trend of rhBMP-2 treated groups to form a larger bone callus than the AV-ADMSCs treated groups. This finding correlated with H&E and SOFG images showing that while rhBMP-2 generated prominent callus formation, the new bone had thin outer cortices and bone tissue within the defect was full of fine, woven trabecular bone interspersed with bone marrow; there was no discernible difference between FK506 treated and untreated animals in groups 7 and 8 (Fig. 3a–d). AV.AD-MSCs, in contrast, produced a more compact bone tissue within the defect, with thick trabeculae, outer cortices fading into the original bone and an almost tubular bone marrow when observed both in μCT and histology images (Fig. 3a–e). All animals in group 5 that did not heal underwent histological analysis, showing areas of new bone tissue filling the defect area with both fibrous and cartilaginous tissue in between the new bone formation (Fig. 3a).

BV/TV ratio of the intact femora was found to be significantly higher when compared to any of the treatment groups that healed the defect (groups 6–8) (*p* < 0.05), except for group 5 where only 3 out of the 9 animals healed the defect. Of all the groups that consistently healed the defect, group 6 had the highest BV/TV ratio (55.2 % ± 14.8) followed by group 7 (44.07 % ± 13) (*p* = 0.144) and group 8 (33.92 % ± 7.07) (*p* = 0.0001). No difference was observed in between groups 7 and 8 (*p* = 0.248) (Fig. 4c).

### Torsional testing of healed bones at 8 weeks

Because BMP-2 treated animals were the only groups to heal the bone defect, only 27 of the 79 operated and harvested femora were able to be used for biomechanical testing. Due to the low number of group 5 animals that healed the defect (*n* = 3), these samples were used for histology because there were insufficient samples to provide statistical power for biomechanical testing (Table 2). All the healed femora failed at the defect site suggesting the newly formed bone to be of lesser quality than the normal bone proximal or distal to the defect. Some samples had fracture lines extending to the proximal and/or distal normal bone without a discernible pattern between groups, with failure being achieved slowly and sometimes only evident when observing the data curve on the software. In contrast, all of the contralateral, intact femora presented with spiral fracture patterns that spanned the diaphysis with little variation between samples and failure progressing from normal, continuous bone to fracture abruptly, accompanied by a loud snap.

**Fig. 5.**
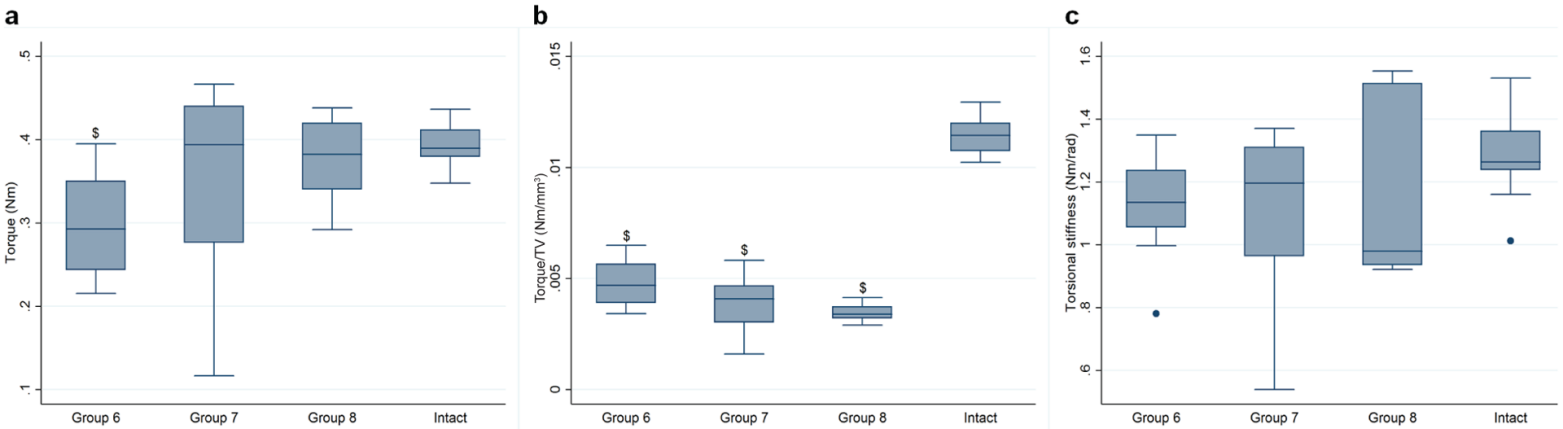
Mechanical properties of harvested femora 8 weeks after various treatments in the presence or absence of systemic FK506 treatment. Treated and untreated femora were harvested from rats 8 weeks after surgery. Both ends of each femur were embedded inside a metal fixture, fixed and torsion was applied to one end until failure occurred. (**a**) Torque to failure. (**b**) Torque to failure normalised by TV. (**c**) Torsional stiffness. Treatment groups were compared to contralateral, unoperated, intact femora. Statistical difference between groups is shown when *p* values are < 0.05. $ denotes statistical difference when compared to intact femora. Data are presented in box and whisker plots representing the interquartile range as a box, the median as the horizontal bar in the box, and the upper and lower adjacent value as whiskers.

Raw torque to failure values showed both rhBMP-2 treated groups to be similar in strength to the contralateral, untreated femora, with group 7 presenting with wider standard deviation than group 8, while femora from group 6 appeared slightly, but statistically less resistant to failure when compared to intact femora (*p* = 0.033) but not to either group 7 (*p* = 0.866) or group 8 (*p* = 0.191) (Fig. 5a). Since callus size is an important variable on this test, data were normalised to their respective TV values obtained from μCT analysis. When normalised in this fashion, all femora in the treated groups were observed to be significantly less resistant to torque than the intact femora (*p* = 0.0001) (Fig. 5b). There was no statistical difference between groups 6, 7 and 8; although it appeared as if group 8 were less resistant to torque than group 6 when the callus size was taken into account, statistical significance was not found (*p* = 0.052). No statistical difference between the groups was detected in raw torsional stiffness values.

## Discussion

This project was triggered by earlier research in this laboratory suggesting that immunosuppression enhanced the healing of critical sized osseous defects when BMP-2 was delivered by adenovirally transduced muscle grafts (De la Vega *et al*., 2018; Liu *et al*., 2015). However, the data from the present study argue that FK506 has very little effect on the healing of critical size, diaphyseal defects in response to BMP-2 as such in the rat femur. Accordingly, FK506 did not influence the healing response of rat critical size defects implanted with rhBMP-2 and had no effect on empty defects.

Nevertheless, the present data show that FK506 did improve healing induced by syngeneic MSCs transduced with a first-generation adenovirus vector carrying cDNA encoding BMP-2. Cells transduced with first generation adenovirus vectors express low amounts of adenoviral proteins which are highly antigenic (Yang *et al.*, 1994), suggesting that the stimulatory effects of FK506 reflect inhibition of an immune response to the transduced cells. However, it cannot be ruled out that FK506 is enhancing the osteogenic properties of the MSCs by stimulating BMP receptor signalling. Investigation of immune responses to adenoviral antigens, their effects on the survival of the implanted cells and the role of these responses in suppressing bone healing are needed to clarify this matter.

The sensitivity of bone healing in this model to immune activation was previously indicated by studies noting enhanced healing using genetically modified muscle grafts from syngeneic, as opposed to non-syngeneic, donor rats (Evans *et al.*, 2009). These conclusions are consistent with the finding that certain sub-sets of T-cells inhibit the regeneration of calvarial defects in mice by autologous MSCs delivered on a ceramic scaffold (Liu *et al.*, 2011). Inhibition is mediated by interferon-γ and tumour necrosis factor-α produced by specific types of T-cells. These cytokines block the differentiation of MSCs into osteoblasts and also trigger their apoptosis.

The dose of rhBMP-2 was also based on previous studies from our group and others that have also used the collagen type I sponge as a delivery vehicle (Liu *et al.*, 2016). Even though the rhBMP-2 dose of 11 μg has been shown to bridge the defect in this rat model by radiological criteria, the quality of the bridged bone is poor. In particular, bridging is achieved by large calluses with thin cortices and wispy trabeculae with characteristically low BV and high TV. The primary aim of the present study was to determine whether FK506 could improve the quality of the regenerate and thus increase the BV, resulting in improved torque to failure results. Improved quality of the regenerated bone using similar doses of BMP-2 has been achieved when other systemic agents (Tinsley *et al.*, 2015) or mechanical environments have been used (Glatt *et al.*, 2012).

Several publications report healing of this type of rat femoral defect using lower doses of rhBMP-2 (Boerckel *et al.*, 2011; Brown *et al.*, 2011; Zara *et al.*, 2011) while using a wide variety of scaffolds different from the one that was used in the current study. The collagen sponge used in the present study was selected because it is the same as that used in the Infuse^®^ product, already being used in the clinic. To determine whether alternative scaffolds would provide a superior matrix was not an aim of this investigation. Likewise, fibrin is commonly used to encapsulate cells for implantation in experiments such as these and it was not the intention to seek to improve upon this. The reason for studying genetically modified cells as well as rhBMP-2 lies with the long-term goal of developing a gene therapy for bone healing. This is based on the hypothesis that gene delivery will provide sustained expression of BMP-2 in a focal manner and thereby provide superior bone healing with less adverse side effects, such as ectopic bone formation.

FK506 failed to demonstrate any obvious ability to initiate or potentiate bone healing at doses similar to those used clinically. In the absence of a robust, reproducible, stimulatory effect of FK506 on bone healing in rodents, it is difficult to foresee a clinical role in treating human fractures and segmental defects, especially as trauma patients are often immunosuppressed already as a result of their injuries (Islam *et al.*, 2016). Although FK506 is FDA-approved, its use is associated with adverse side effects including osteopenia and osteoporosis (Kulak *et al*., 2012). This effect also occurs in rats, where trabecular volume is reduced even in animals where FK506 promotes ectopic osteogenesis on a collagen matrix elsewhere in the body (Voggenreiter *et al*., 2000).

The histological images in Fig. 3 confirm that, although rhBMP-2 is able to heal critical sized defects in the rat femur, the quality of the new bone is poor with a large callus, thin cortices and wispy trabeculae. In contrast, new bone formed in response to BMP-2 synthesised endogenously by genetically modified cells has smaller callus, much better cortication and robust reconstitution of bone marrow. Femora treated with rhBMP-2 were able to achieve similar torsional strength to that of intact femora only by forming a very large callus as evidenced by μCT results, where AV.AD-MSCs and rhBMP-2 treated animals had similar BV but both rhBMP-2 treated groups had a significantly higher defect TV. When an attempt was made to normalise torsional strength results to the callus size a reversion of values was observed, with the AV.AD-MSCs treated femora reflecting higher torque to failure per cm^3^ values just shy of statistical significance (*p* = 0.052) which could potentially indicate that the study was underpowered for this variable. These large bone calluses, although innocuous to the current particular bone defect model, could potentially generate adverse events in a clinical setting, especially for defects in the vicinity of neurovascular bundles.

The amounts of BMP-2 needed for bone healing by genetically modified cells is 2–3 orders of magnitude less than those needed by rhBMP-2, and much closer to those present physiologically. The data from the present study suggest that first generation adenovirus is not an ideal vector for cell modification in this context because of the immune reactions it provokes. Other vectors, however, are available for this approach to bone healing.

## Figures and Tables

**Table 1.** Study design. Groups of rats were assigned to experimental groups as indicated. FK506 at 1 mg/kg/d was released *via* an osmotic pump systemically for 4 weeks in half the animals. Empty femoral defects were used as negative controls. AD-MSCs were harvested from donor rats and expanded *in vitro* prior to encapsulation in fibrin gels at a density of 4 × 10^6^ cells/gel. Cells were encapsulated either non-transduced or after transduction with a first generation adenovirus encoding human BMP-2. Recombinant human BMP-2 protein was loaded onto an absorbable collagen sponge. All animals were subjected to x-rays at days 10, 28, 42, 56 and euthanised after 8 weeks. All rats underwent μCT analysis. Healing of the defects was analysed by μCT (all animals) and biomechanical testing of bridged defects in 8 rats/group. The remainder were used for histology. Any unhealed defect was also used for histology.

Group	Rat number	Defect treatment	FK506	BMP-2	
				recombinant	cDNA
1	10	Empty	−	−	−
2	10	Empty	+	−	−
3	10	AD-MSCs	−	−	−
4	10	AD-MSCs	+	−	−
5	10	AV.AD-MSCs	−	−	+
6	10	AV.AD-MSCs	+	−	+
7	10	rhBMP-2 (11 μg)	−	+	−
8	10	rhBMP-2 (11 μg)	+	+	−

**Table 2:** Radiologic bridging per group. Summary of radiologic bridging results and end point testing of specimens for each treatment group. One rat in group 5 was euthanised at day 10 because of a femoral fracture.

Group	Insert	Bridging	Histology testing	Biomechanical testing
1	Empty	0/10	10	0
2	Empty + FK506	0/10	10	0
3	AD-MSCs	0/10	10	0
4	AD-MSCs + FK506	0/10	10	0
5	AV.AD-MSC	3/9*	9	0
6	AV.AD-MSC + FK506	10/10	2	8
7	rhBMP-2	10/10	2	8
8	rhBMP-2 + FK506	10/10	2	8

* denotes statistical difference as determined by the Pearson χ^2^ test when comparing bridging results of one treatment group to its equivalent receiving FK506.

## Discussion with reviewers

Reviewer 1: How would you explore the effects of FK506 on the immune response observed in your experimental model?

Authors: As discussed in the paper, we hypothesise that adenovirally transduced cells are destroyed by the immune system and this is alleviated by FK506. To test this, in future work it will be necessary to measure humoral and cell-mediated responses to adenovirus serotype 5 in recipient rats. The former can be measured by an *in vitro* neutralising antibody assay which tests the ability of rat serum to inhibit the ability of adenovirus5 to transduce cells in culture. Cell mediated responses are measured by ELISPOT assays which detect the production of interferon-γ by T-cells exposed to adenoviral antigens. Separately, we will need to show that the implanted cells survive longer in the presence of FK506. It would be interesting also to repeat key experiments from this paper using other viral vectors, such as adeno-associated virus and lentivirus, that do not trigger immune responses to transduced cells.

Nick Willett: Please discuss the rationale and any potential limitations of the doses of FK506, BMP-2 and MSC that were used.

Authors: Doses were selected on the basis of prior work. This imposes the limitation that other doses were not evaluated, leaving open the possibility that these could provide different results.
